# Minimally invasive biomarkers for triaging lung nodules—challenges and future perspectives

**DOI:** 10.1007/s10555-025-10247-5

**Published:** 2025-01-31

**Authors:** Waqar Ahmed Afridi, Samandra Hernandez Picos, Juliana Muller Bark, Danyelle Assis Ferreira Stamoudis, Sarju Vasani, Darryl Irwin, David Fielding, Chamindie Punyadeera

**Affiliations:** 1 Saliva and Liquid Biopsy Translational Laboratory, Institute for Biomedicine and Glycomics (IBG), Griffith University, Brisbane, 4111 Australia; 2 Virtual University of Pakistan, Islamabad, 44000 Pakistan; 3 Department of Otolaryngology, Royal Brisbane and Women’s Hospital, Herston, 4006 Australia; 4 The Agena Biosciences, Bowen Hills, Brisbane, 4006 Australia; 5 The Royal Brisbane and Women’s Hospital, Herston, Brisbane, 4006 Australia

**Keywords:** Lung nodules, Biomarkers, Early diagnosis, Minimally invasive biopsies, Artificial intelligence, Radiomics

## Abstract

CT chest scans are commonly performed worldwide, either in routine clinical practice for a wide range of indications or as part of lung cancer screening programs. Many of these scans detect lung nodules, which are small, rounded opacities measuring 8–30 mm. While the concern about nodules is that they may represent early lung cancer, in screening programs, only 1% of such nodules turn out to be cancer. This leads to a series of complex decisions and, at times, unnecessary biopsies for nodules that are ultimately determined to be benign. Additionally, patients may be anxious about the status of detected lung nodules. The high rate of false positive lung nodule detections has driven advancements in biomarker-based research aimed at triaging lung nodules (benign versus malignant) to identify truly malignant nodules better. Biomarkers found in biofluids and breath hold promise owing to their minimally invasive sampling methods, ease of use, and cost-effectiveness. Although several biomarkers have demonstrated clinical utility, their sensitivity and specificity are still relatively low. Combining multiple biomarkers could enhance the characterisation of small pulmonary nodules by addressing the limitations of individual biomarkers. This approach may help reduce unnecessary invasive procedures and accelerate diagnosis in the future. This review offers a thorough overview of emerging minimally invasive biomarkers for triaging lung nodules, emphasising key challenges and proposing potential solutions for biomarker-based nodule differentiation. It focuses on diagnosis rather than screening, analysing research published primarily in the past five years with some exceptions. The incorporation of biomarkers into clinical practice will facilitate the early detection of malignant nodules, leading to timely interventions and improved outcomes. Further efforts are needed to increase the cost-effectiveness and practicality of many of these applications in clinical settings. However, the range of technologies is advancing rapidly, and they may soon be implemented in clinics in the near future.

**Graphical abstract:**

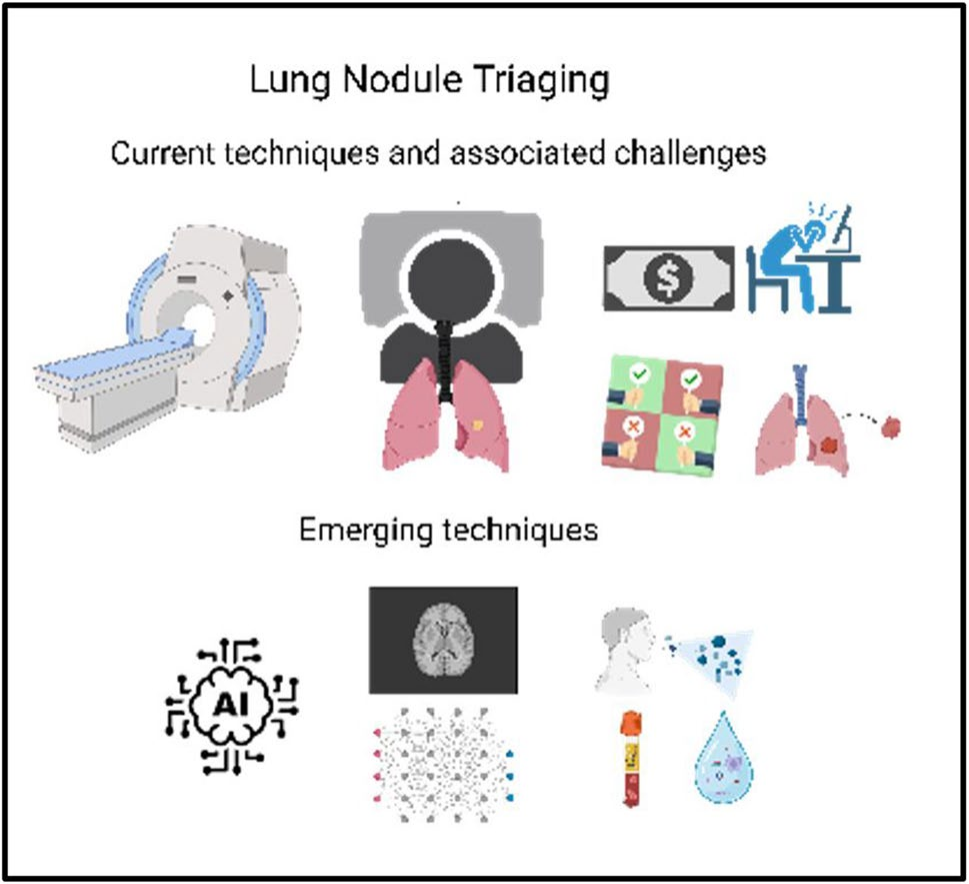

## Introduction

Lung nodules are round or oval-shaped densities seen on CT scans. The concern regarding a nodule is that it may be lung cancer. Lung cancer is the primary cause of cancer-related mortality worldwide, with more than 2.2 million new cases diagnosed and 1.8 million deaths in 2020. The number of annual cases of lung cancer is expected to reach 3.8 million in 2050 [1]. Smoking is the primary cause of lung cancer. However, a significant number of lung cancer cases (15–25%) are also observed in patients who have never smoked [2]. Lung cancer can develop as single or multiple lung nodules and subsequently spread to regional thoracic lymph nodes and other organs outside the thorax. Only 15% of new lung cancer patients are diagnosed at an early stage because of the asymptomatic nature of the disease in its initial phases [3]. Unfortunately, most patients are diagnosed at advanced stages (III or IV) when effective treatment options are limited, leading to poor survival outcomes. Patients diagnosed with stage IA lung cancer have a > 75% 5-year survival rate, whereas patients diagnosed at a late stage have a 5% survival rate [4].

Lung nodules are currently detected either by CT screening programs or as incidental findings on non-screening CT scans. Lung cancer screening via low-dose CT (LDCT) is now part of the medical landscape following two landmark trials — the National Lung Screening Trial (NLST) and the recent Dutch-Belgian Randomised Lung Cancer Screening Trial ( NELSON) studies — leading to a reduction in lung cancer mortality by 20% [5]. The U.S. Preventive Services Task Force (USPSTF) recommends yearly LDCT screening for high-risk individuals between 50 and 80 years old with a smoking history of at least 20 packs per year who are current smokers or have quit within the past 15 years [6]. Lung nodules are evaluated for size, density, shape, and interval changes. As outlined below, the Lung Imaging Reporting and Data System (Lung-RADS) and Pan-Canadian Early Detection of Lung Cancer (PanCan) criteria have significantly improved biopsy decision-making around CT-detected nodules, but clinical studies regarding the clinical outcomes of using these tools are ongoing.

With CT-detected nodules, a high percentage (up to 96%) of the nodules detected through LDCT are benign, and unnecessary biopsy procedures may eventuate for benign nodules [7]. Follow-up of small indeterminate nodules with repeated CT scans may cause unwanted stress to an individual. Second, many are present in patients with underlying lung conditions, such as chronic obstructive pulmonary disease (COPD), rendering biopsies more challenging, and adverse biopsy outcomes can occur, including when a biopsy is performed on a nodule that is ultimately actually benign. There is the possibility that nodule interpretations can significantly vary and lead to imprecise decisions [8]. In response to these challenges, strategies for handling CT-detected nodules based on nodule size and other clinical parameters, including the Lung-RADS and the PanCan criteria, have been developed [9, 10]. Nonetheless, there is still the issue that patients can still ultimately have biopsies for benign nodules.

Biomarkers can be applied to CT nodule detection and characterisation in three ways [11, 12]. First, they can be used in high-risk individuals within a screening program for the early detection of disease. In this context, biomarkers are typically combined with the analysis of CT findings, although in some cases, they can serve as a standalone screening modality independent of CT [13]. Second, biomarkers could be used to identify higher-risk groups—enhancing clinical criteria for inclusion in a screening program—allowing patients to be classified as higher risk based on biomarkers rather than clinical factors alone [14]. Third, biomarkers can be used after CT nodules are detected to distinguish between benign and malignant nodules, potentially reducing the need for unnecessary biopsies, especially for lesions that are ultimately found to be benign. This review focuses on the third aspect of biomarker testing. Specifically, we examine how biomarkers—especially groups of biomarkers—can be integrated with radiology rather than relying on a single biomarker to provide all the answers. In Sect. 3, we explore the development of a multiplexed approach to nodule characterisation.

Therefore, innovative tools to distinguish malignant from benign nodules effectively are needed. These biomarkers are present in biofluids (blood, saliva, urine, and effusion) [15] and breath [16]. Body fluids and breath biopsy-based biomarkers have great potential because of their easy accessibility, minimally invasive nature, suitability for both inpatient and outpatient testing, reported cost-effectiveness, and potential for repeated sampling for disease monitoring [17]. Currently, biomarkers such as circulating tumour cells (CTCs), exosomes, DNA methylation (DNAm), tumour-derived circulating nucleic acids (cell-free DNA, cfDNA), microRNAs (miRNAs), noncoding RNAs (ncRNAs), and volatile organic compounds (VOCs) are applied in a research context for the evaluation of CT-detected nodules, enabling the potential for triaging biopsy decisions and potentially facilitating the targeting of biopsies to patients who are more likely to have cancer [18].

In this comprehensive review, we analysed the most recent studies that have used biomarkers for triaging lung nodules and addressed the associated challenges. While studies with individual biomarkers often yield encouraging results, none have featured sufficient for “prime time” use individually. Nevertheless, numerous new potential biomarker candidates have been published, setting the stage for developing biomarker combinations that can be used in the clinic for patients with CT-detected nodules in the future. We focused primarily on the diagnosis rather than the screening of lung nodules. Additionally, we offer insights and recommendations for future research to develop strategies for incorporating biomarker research in nodule triage.

## Current techniques for characterising lung nodules and their limitations

### Lung nodules

#### Defining lung nodules

**Fig. 1:**
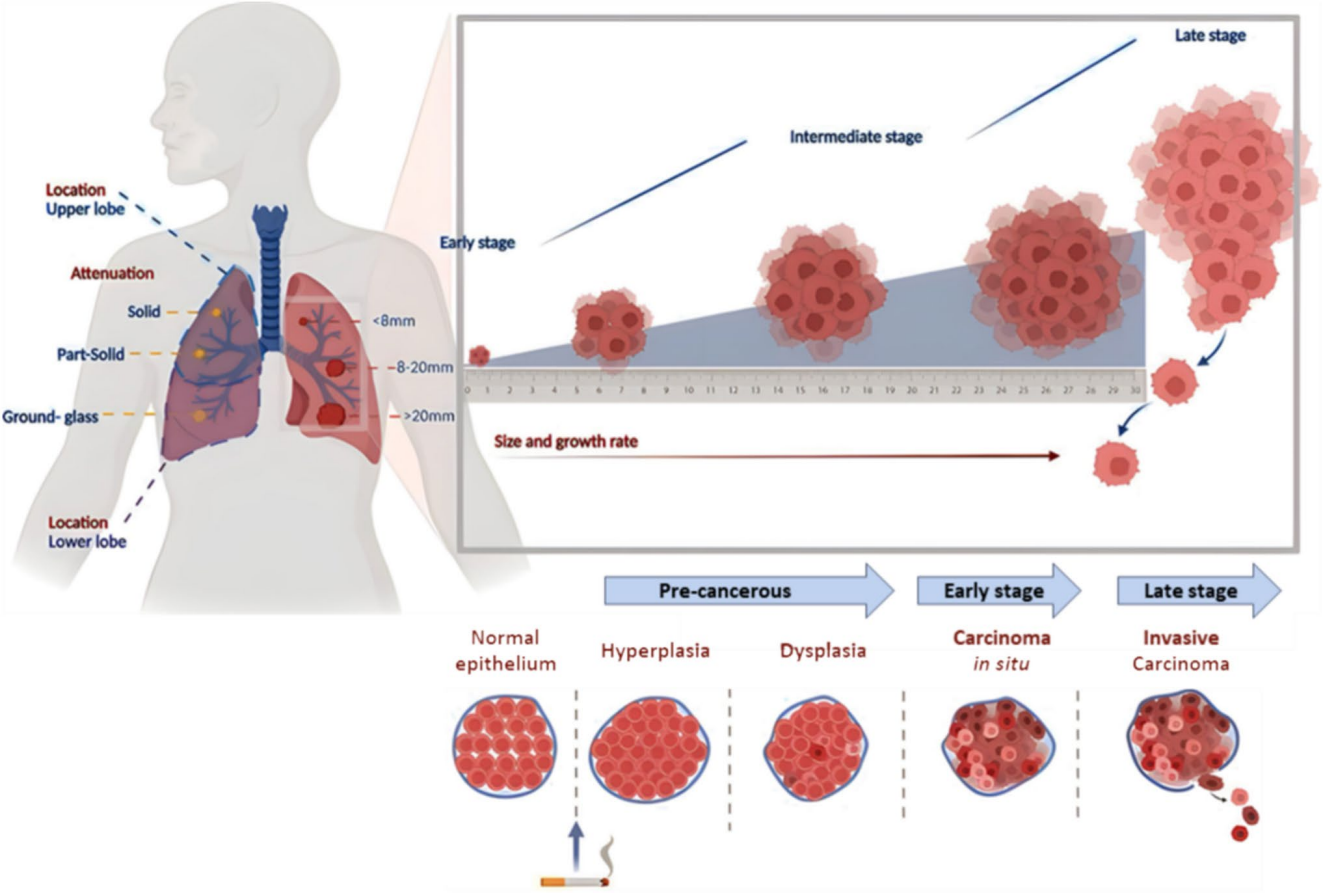
A visual depiction of lung cancer progression from the normal epithelium to premalignant stages and malignancy. Solid nodule growth is depicted. Morphological changes in the bronchial epithelium, including hyperplasia, metaplasia, dysplasia, carcinoma *in situ* (CIS), invasive cancer, and metastatic cancer, lead to progressively worse outcomes. For ground glass opacities (GGOs), the current lung nodule guidelines and risk prediction models suggest that the solid component of a GGO nodule indicates its malignancy grade. Created with BioRender.com

A lung nodule is a small, rounded opacity (< 30 mm) surrounded by lung tissue. Rounded tumours greater than 30 mm in diameter are called lung masses and should be considered cancerous unless histologically proven otherwise [19].To date, a limited number of nodule features (size, growth rate, shape, location, and density) have been used to evaluate the risk of malignancy (Fig. 1).

#### Solid nodules and ground glass opacities

Lung nodules can be divided into (i) solid nodules (small, < 8 mm and large, > 8 mm), the most common type, and (ii) subsolid [20]. Subsolid nodules are classified into two subtypes according to their density: ground-glass (without a solid component) and part-solid (with a solid component) [21]. Subsolid nodules are challenging because they carry a high degree of malignancy risk but have a slow growth rate [22]. They have a 10–50% probability of malignancy if they persist for over three months and exceed 10 mm in diameter [23]. Additionally, the size of the solid component impacts how lung nodules are managed. A larger percentage of the solid part is associated with a greater likelihood of malignancy [22].

#### Anatomical location

In addition to the degree of nodule attenuation and size, the location also determines the likelihood of malignancy. In high-risk populations undergoing screening, up to 45% of all cancerous nodules are in the upper lobes, particularly the right upper lobe [24, 25].

### Radiologic methods

#### CT scanning including low-dose CT screening

There is no specific CT feature that can confidently rule out a lung nodule as being malignant. However, several factors are associated with malignancy. The first consideration is the size and growth rate of the lung nodules. The size of the nodule remains the most critical factor determining the likelihood of malignancy. With increased size, the probability of malignancy of lung nodules also increases. With increasing size, the probability of malignancy increases from 1% for smaller lesions (< 6 mm) to 64% for lesions > 20 mm in size [26]. Cancerous lung nodules grow relatively quickly, usually with a volume-doubling time (VDT) of 100 or 400 days. According to observations of the Dutch-Belgian Randomised Lung Cancer Screening Trial (Dutch acronym: NELSON study), during baseline screening, 2.3% of patients' lung nodules had a VDT of less than 400 days [27]. However, Zhang et al. [28] demonstrated that a VDT of less than 400 days is not an exclusive predictor of malignant nodules. In their study, 16% of the rapidly growing nodules were benign lesions.

Another issue is that false-positive results (i.e., scan findings leading to additional examinations where the final diagnosis is benign) can lead to intrusive and unnecessary procedures, followed by increased anxiety for patients [29]. The false-positive rates range from 7.9 to 49.3% [30]. In the NLST study, the false-positive rate was 96.4% according to baseline screening via LDCT. However, 9% of patients undergo invasive biopsy procedures [31]. Additionally, false-negative results can occur: a false negative outcome indicates that a person with a cancerous lung nodule receives an incorrect negative result [32]. False-negative results increase the potential for delayed diagnosis and may result in a worse prognosis of patients with lung cancer because of stage shifts. False-negative results are reported in 8% to 15% of patients diagnosed through screening trials [33]. Handling nodules detected during LDCT screening poses a complex challenge that necessitates the expertise of radiologists who can consistently interpret the images via standardised protocols. Implementing specific guidelines for health practitioners can help reduce the occurrence of overdiagnosis, false-positive results and intraobserver variability [34].

Excellent clinical tools (Lung-RADS and PanCan / Brock) combine information from clinical history (age, pack-years, history of COPD or asbestos exposure) and LDCT (nodule size and location, and presence of pulmonary emphysema) to further aid in the decision-making of lung nodules discovered at baseline via LDCT screening [35–37]. In 2014, the American College of Radiology (ACR) introduced Lung-RADS v1.0 to standardise CT screening reporting, offer uniform management guidelines, and facilitate the monitoring of patient outcomes after screening [38]. Lung-RADS was upgraded to v1.1 in 2019, which included new size criteria for nonsolid nodules, updated measurement criteria, and classification criteria for perifissural nodules [35]. The latest version, Lung-RADS v2022, released in November 2022, provides additional guidelines for classifying and managing findings related to the airways during LCS, which considers location, morphology, number of findings, and persistence at follow-up imaging [9].

The Lung-RADS is used to define clinical risk into four levels [37], with scores of 0, 1 and 2 indicating a < 1% chance of malignancy, increasing to category 4A (5–15% chance of malignancy) and 4B (including nodules >  = 15 mm) with a > 15% chance of malignancy. Patients in categories 1 and 2 will have repeat CT at 12 months, Category 3 at six months, and Category 4A at three months (with an option for PET/CT then), whereas 4B patients recommend immediate PET/CT and/or tissue sampling. The stability of nodules at 3 and 12 months is an important factor in down-classifying nodules via the Lung-RADS. An evaluation of this strategy from a 2015 study [39] revealed an increased positive predictive value of an LDCT screening cohort by a factor of 2.5. It reduced the overall positive rate on the basis of CT images from 28 to 11%. The PanCan study [36] utilised the multifactor clinical Brock score, leading to clinical recommendations based on cancer probabilities, known as the PanCan model. The British Thoracic Society (BTS) guidelines for pulmonary nodules recommend this risk prediction score. Here, the recommendation for a nodule Brock score > 10% ( Grade D evidence) is “Discuss the options of observation with repeat CT, CT-guided biopsy, or resection/non-surgical treatment with the patient where the risk of malignancy is approximately > 10%; consider factors such as age, comorbidities and risk of surgery”. A 2015 validation study [10] on the PanCan model revealed an AUC of 0.83–0.87 in a screening cohort of 1152 nodules. Kim et al. [40] compared the Lung-RADS and PanCan models in a screening cohort of 13150 patients and demonstrated similar discrimination performance on baseline CT (AUCs of 0.96 and 0.95, respectively). For a 10% risk, the sensitivity and specificity of the PanCan model were 83% and 96%, whereas those of the Lung-RADS model were 88% and 93%, respectively. Although the Brock model is highly accurate for diagnosing lung nodules, it requires extensive input information—six nodule-level and three participant-level factors—that may not always be readily available when interpreting screening CT scans. A simpler model with fewer inputs would be easier for physicians to practice and might be more likely to be adopted in everyday clinical practice [41].

Lung-RADS has recently been updated; it is now version v2022 [42]. Important updates include nodule growth parameters (defined as > 1.5 mm mean diameter increase within a 12-month interval), classifying nodules as rapid or slow growing (growing by < 1.5 mm in a 12-month interval), and greater clarity on the role of CT volumetrics (ongoing studies are needed to benefit over conventional size measurements).

#### Positron emission tomography/computed tomography (PET/CT)

Positron emission tomography using Fludeoxyglucose F18 (FDG) (PET) has the potential to enhance lung nodule triaging [43]. In 1996, Gupta et al. [44] published one of the earliest studies in which FDG PET scans were used to differentiate benign and malignant solitary pulmonary nodules (SPNs) and reported greater accuracy than conventional imaging methods did. Nodules with low FDG uptake are most likely benign (such as granulomas or hamartomas), with a negative predictive value of 92%, whereas nodules with high FDG uptake are more likely malignant, with a high positive predictive value of 90% [45]. A study conducted by Hadique et al. [5] supported the use of FDG PET/CT as an effective tool for evaluating lung nodules, leading to more accurate diagnoses and better patient outcomes. These results could assist physicians in choosing between regular monitoring and more invasive procedures, such as biopsies.

Herder et al. [46] combined clinical assessment with a four-class PET/CT result to obtain a combined risk score, such that a risk of > 70% should lead to treatment, whereas a score of < 10% suggests ongoing conservative management. Presently, BTS guidelines for nodule evaluation suggest the use of this index to improve decision-making further [47].

The assessment of small nodules of approximately 5–8 mm can be hampered by low FDG uptake [48]. Additionally, normal tissues can naturally absorb FDG, leading to misinterpretations and false positive and false negative results [43, 45]. Despite advancements in graphics processing units (GPUs), the computational demands of processing 3D datasets remain significant. Although future technological advancements may render this a less pressing issue, current limitations in computational power hinder the efficient analysis of multiple large-scale 3D datasets encompassing entire anatomical regions captured by a PET/CT scan [49].

Overall, PET/CT is a well-established tool for nodule evaluation that can influence biopsy decision-making, but there are recognised limitations due to very small nodule size (8 – 9 mm) and false positives due to inflammatory nodules [5] and the issue of incidental detection of non-pulmonary abnormalities, which require investigation on their own right [50].

### Noninvasive biomarkers

#### Serum tumour markers

Lung cancer can be divided into two broad groups based on histopathology: non-small cell carcinoma (NSCLC) and small cell carcinoma (SCLC). NSCLC is the predominant target of screening techniques and is divided into adenocarcinoma (AC), squamous cell carcinoma (SCC) and large cell (undifferentiated) carcinoma). Among these, ACs arise from mucus-producing cells, typically occupying the periphery of the lung and are the most common cancer type detected by CT screening because the lung parenchyma is well visualised. In contrast, SCCs arise in squamous epithelial cells and tend to occupy a more central location on CT scans. Small cell carcinomas arise from neuroendocrine cells in the lung parenchyma (in contrast to NSCLC, which arises from epithelial cells) and grow very rapidly [51].

Currently, serum biomarkers such as cytokeratin 19 fragment (CYFRA21-1), pro-gastrin releasing peptide (ProGRP), carcinoembryonic antigen (CEA), neuron-specific enolase (NSE), and squamous cell carcinoma-associated antigen (SCCA) have shown potential clinical utility for diagnosing lung cancer [52]. While there is promise at this time, serum biomarkers have not been included in screening program guidelines. Notably, while elevated levels of specific serum markers can be associated with lung cancer, and some may even exhibit correlations with specific histological subtypes, their sensitivity and specificity as standalone diagnostic tools are inadequate [53].

In a study investigating the diagnostic utility of tumour markers for solitary pulmonary lesions, CEA showed a sensitivity of 27.2% and an accuracy of 40.4%, and CYFRA21-1 demonstrated the highest specificity (100%) for detecting lung cancer [54]. Fu et al. [55] also demonstrated that CYFRA21-1 is a well-explored biomarker for NSCLC. They reported that the sensitivity and specificity of the CYFRA21-1 tests for detecting squamous cell carcinoma were 72% and 94%, respectively. Pro-gastrin-releasing peptide (pro-GRP), the precursor to gastrin-releasing peptide (GRP), is another established tumour marker. However, owing to the short half-life of GRP (two minutes), the use of pro-GRP has recently increased [56]. Rafael Molina et al. [57] reported the diagnostic performance of a panel of tumour markers, including CYFRA21-1, ProGRP, CEA, NSE, and SCCA, for lung cancer diagnosis. These findings revealed that CYFRA21-1 had the highest sensitivity (56.1%), followed by CEA (56.5%) and NSE (19.1%). In terms of specificity, all five markers exhibited promising results, exceeding 90% (CYFRA21-1: 96.1%, ProGRP: 95.2%, CEA: 93.5%, NSE: 99.5%, and SCCA: 97.8%). Notably, these markers retain high specificity even for nodules smaller than 30 mm. Although combining multiple tumour markers may improve diagnostic sensitivity, this approach can potentially come at the expense of specificity and incur increased healthcare costs. Identifying the optimal combination of markers is crucial for maximising diagnostic efficacy and minimising the financial burden on patients and healthcare systems. As prior reports suggested, cost-effectiveness analyses are warranted to evaluate the utility of combining tumour markers in diagnosing lung cancer [58]. However, the current serum biomarkers do not meet the clinical sensitivity and specificity for inclusion in standard guidelines.

## Emerging techniques for characterising individual biomarkers of lung nodules

### Artificial intelligence (AI) and radiomic analysis

Numerous AI-based radiologic tools (radiomics) have been developed, particularly for lung segmentation, nodule detection, and characterisation [59]. Importantly, radiomics is noninvasive and suitable for automatic serial monitoring. Artificial intelligence (AI) advances in radiology with respect to nodules have come in 2 forms [60–62]. Initially, computer-aided detection (CAD) was used to screen the entire CT image for nodules.

Computer-aided detection of lung nodules is used to screen a CT image for the presence and location of nodules [62]. Various CAD tools have been commercially available for pulmonary nodule detection since the early 2000s. CAD tools are software applications that use AI-based algorithms that excel at automatically recognising complex patterns in imaging data and providing quantitative, rather than qualitative, assessments of radiographic characteristics to aid radiologists. These approaches necessitate reduced radiologist time and often yield superior detection results [63]. Several CAD tools with relatively low false-positive results per examination have recently been developed because of the rapid rise of deep learning in recent years and the accessibility of large, annotated lung nodule datasets [64]. In general, the CAD system produced results comparable to those of radiologist review alone. However, prospective clinical studies indicate that when combined with expert radiology review, computerised detection enhances the identification of suspicious nodules. [65–67].

More recent AI advances for nodules have focussed on morphology and texture analysis. AI has been applied to basic nodule morphologic criteria, including volumetric analysis, growth characterisation and better detail related to nodule margins [63, 68]. These are all visible or observable traits of a nodule that can nonetheless be significantly better characterised by AI techniques. Second, invisible or “agnostic” traits — purely electronic in nature — can be accumulated through the analysis of thousands of nodules with known pathology tendencies [69]. These AI tools can be used to evaluate nodules once they have been located by CAD to score the nodule for benign versus malignant [70].

Through mathematical extraction of the spatial distribution of signal intensities and pixel interrelationships, radiomics quantifies textural data via AI techniques [71]. This form of AI has very significantly advanced nodule characterisation and holds significant future potential. As large CT datasets of nodules with known pathology continue to accumulate, they are expected to undergo continuous improvement. For example, a 2020 study of indeterminate pulmonary nodules reported an AI platform built from data obtained from NSLT CT scans: 14,761 benign nodules from 5,972 patients and 932 malignant nodules from 575 patients [72]. The results were compared with those of clinical risk predictors. The receiver operating characteristic(ROC) curves had AUC values of 83.5% and 91.9% for the external validation cohort, whereas they were 78.1% and 81.9%, respectively, for a commonly used clinical risk model for incidental nodules. This led to correct changes in the deemed clinical risk for low versus high risk in 34% of cases as a rule-in test and 58% of cases as a rule-out test. The authors agreed that further study is needed to determine exactly how this impacts clinical biopsy decision-making. Radiomics also offers several logistical advantages. For example, it provides real-time results and could reduce the need for invasive procedures. Furthermore, compared with the limitations of standard biopsy, radiographic images provide complete and more comprehensive details. The classification of lung nodules from CT data in combination with AI is still challenging due to the heterogeneity of lung nodules, the high degree of similarity between benign and malignant nodules and their similarity to their surrounding tissue [73]. Furthermore, several ML-based approaches have demonstrated a partial ability to differentiate between benign and malignant lesions owing to the lack of available data for benign lung nodules [74].

Radiomics has the potential to reveal the future clinical behaviours of a tumour based on features such as tumour heterogeneity and provide insight into treatment responses [75, 76]. A recent study from Chung Shan Medical University (NCT06282068) evaluated AI in assessing the malignant potential of ground glass opacities [77].

Currently, there is limited use of nodule AI in the clinic, but it is expected to become part of the clinical landscape of nodule evaluation within the next few years as more large datasets are analysed. For example, one such recent study evaluated 1397 patients via a convolutional neural network, resulting in only 1 false negative (0.4% of cancers) compared with the Brock model, where there was a 2.5% false negative rate [78].

A recent large-scale study from China highlighted the potential of advanced AI radiomic tools. [79]. They described the Chinese Lung RADS approach (C-lung-RADS); some 45,000 nodule cases were analysed. Initially, a 3-class system was developed based on accepted radiological criteria for nodule risk (with some AI input). This resulted in an AUC of 0.88. Then, progressively, more information was integrated via the agnostic CT texture features along with the clinical features, resulting in an AUC of 0.92. C-Lung-RADS outperformed Lung-RADS v 2022 in a further independent cohort (sensitivity 87% vs 63%).

Radiomics faces several hurdles that must be addressed before it can be translated into clinical practice. Significant challenges include the interpretability of models, the reproducibility of quantitative imaging features, and the sensitivity to variations in image acquisition and reconstruction parameters. Despite promising findings, the field of radiomics suffers from inadequate standardisation and generalizability of results, limiting its clinical applicability. Importantly, certain limitations concerning data quality control, repeatability, reproducibility, generalizability of findings, and model overfitting have emerged, which should be carefully considered when developing and applying radiomic techniques [80].

### Liquid biopsy

**Fig. 2:**
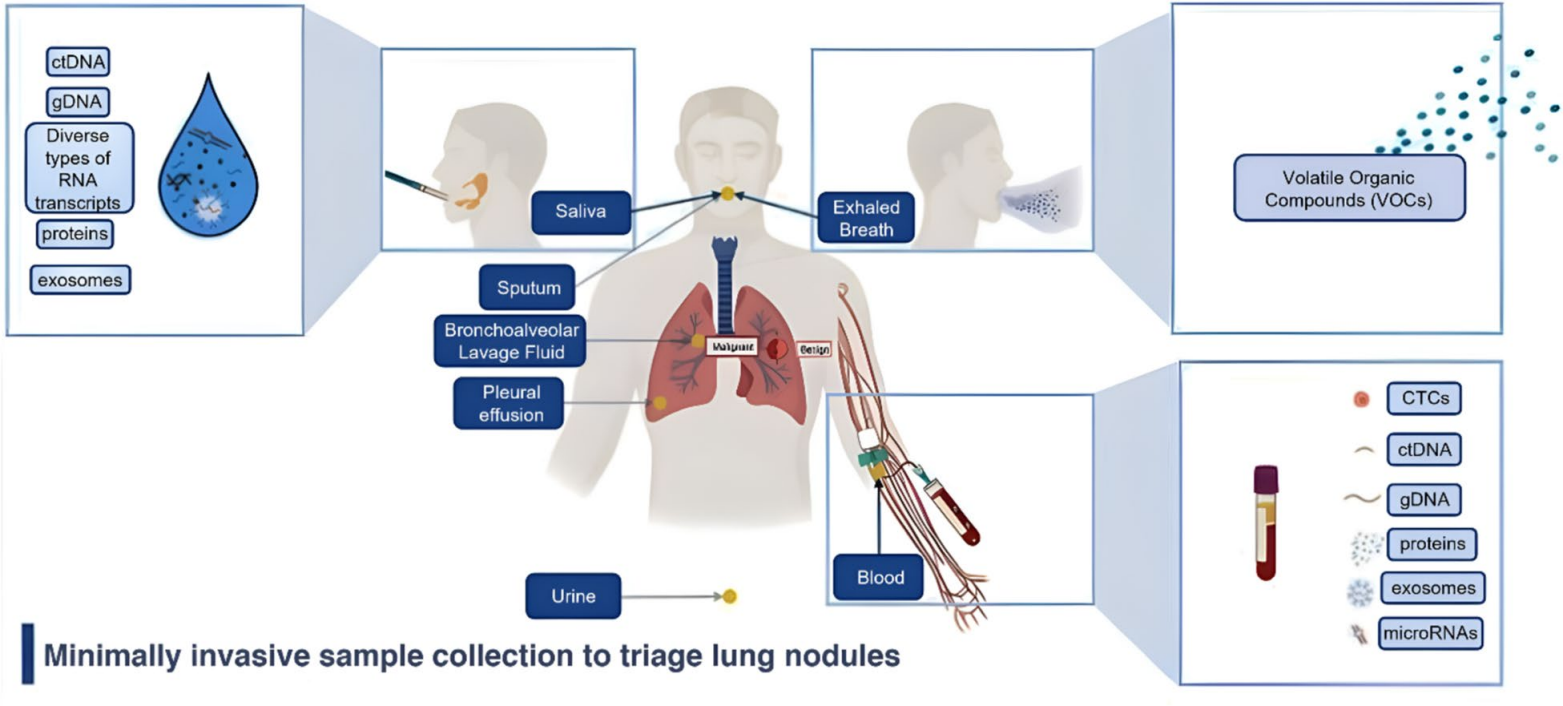
The application of biomarkers via minimally invasive sample collection methods shows promise in differentiating benign from malignant lung nodules. Several body fluids such as blood, saliva, sputum, bronchoalveolar lavage fluid (BALF), pleural effusion, urine, and breath samples (exhaled breath) can be used to enhance biopsy findings or can independently provide insights into the malignant status of lung nodules. Biomarkers such as ctDNA, gDNA, microRNAs, proteins, exosomes, VOCs and circulating tumour cells (CTCs) are found in body fluids and are used for the differential diagnosis of lung nodules. Created with BioRender.com

Liquid biopsy-based biomarkers are emerging as a minimally invasive method to address the temporal and spatial heterogeneity of tumours [81–83] (Fig. 2). According to the National Institute of Health (NIH) [84], a biomarker is an objective measurement or evaluation of a characteristic that indicates normal biological processes, pathogenic processes, or the response to a therapeutic intervention. A valuable biomarker should directly influence clinical decision-making, ultimately leading to better patient care. Despite advancements in biomarker research, only a few have successfully translated to clinical practice. Most biomarkers fail at the clinical utility stage.

For a biomarker to be deemed successful, it should be highly sensitive, specific, and cost-effective and demonstrate its potential clinical utility in the target population [84]. The biomarkers that are present in liquid biopsy include exosomes, microRNAs (miRNAs), circulating tumour cells (CTCs), circulating DNA (cell-free tumour DNA, cfDNA), cell-free tumour RNA (cfRNA), and epigenetic biomarkers (DNA methylation, DNAm). Some of these biomarkers have shown strong performance characteristics and reproducibility, enabling them to accurately reflect patients' tumour changes in real-time [85].

#### Exosomes

Tumour-derived exosomes (TDEs) have emerged as promising diagnostic and prognostic biomarkers for several cancer types and have the potential for early lung cancer detection [86]. Exosomes are small extracellular vesicles (sEVs) that typically range from 30–150 nm in diameter and are characterised by their unique lipid bilayer membrane structure [87–89]. Exosomes contain important biomolecules, such as proteins, RNA, and DNA [90]. Notably, these vesicles are abundantly present in readily available bodily fluids, including blood, saliva, breast milk, and urine [91]. Exosomes participate in cell-to-cell communication and can transfer genetic material from one cell to another, affecting the function of target cells [92]. Kuang et al. [93] investigated plasma sEVs from patients with benign and malignant lung nodules. They reported relatively high levels of fibrinogen beta chain (FGB) and fibrinogen gamma chain (FGG) in sEVs derived from patients with malignant lung nodules. In a similar study, Kuang et al. [94] demonstrated that the use of FGB (sensitivity = 0.628, specificity = 0.800, and AUC = 0.741) or FGG (sensitivity = 0.535, specificity = 0.850, and AUC = 0.659) alone did not achieve the sensitivity required to discriminate malignant from benign lung nodules. However, when FGB and FGG were combined, the sensitivity (0.814) and AUC (0.794) for diagnosing benign and malignant nodules improved compared to using FGB or FGG alone. These results suggest that combining FGB and FGG in plasma exosomes is a more effective marker for differentiating benign from malignant nodules. However, there are several challenges when using exosomes as biomarkers in cancer research. One of the significant challenges is to isolate a sufficient number of sEVs. The most commonly used method is ultracentrifugation, which is considered the most reliable method for separating exosomes. However, this method suffers from impurities, such as contamination with protein aggregates and lipoproteins, making it difficult to accurately measure and analyse the quantity and functionality of proteins [90].

#### Noncoding RNAs

Only a small percentage (approximately 3%) of genetic transcripts are responsible for encoding proteins, whereas the rest are categorised as noncoding RNAs (ncRNAs). The term “coding” refers to RNAs that translate DNA information into proteins, such as mRNAs. In contrast, ncRNAs play different roles, functioning as regulators of gene expression at various levels, including transcription, post-transcription, and epigenetic mechanisms. There are several exceptions to this definition, notably specific ncRNAs that can bind to ribosomes and encode peptides that modulate cellular functions [95].

##### MicroRNAs

MicroRNAs (miRNAs) are small noncoding RNA molecules that typically consist of 18–24 nucleotides and have gained significant recognition as pivotal regulators with diverse functions [96–99]. These miRNAs have become potential early cancer biomarkers because the aberrant expression of miRNAs leads to the development of lung cancer by regulating critical processes such as cell growth, programmed cell death, cell motility, and invasion into surrounding tissues [100]. Cazzoli et al. [101] used blood samples and developed a microRNA panel consisting of miR-378a, miR-379, miR-139-5p, and miR-200b-5p to differentiate benign from malignant lung nodules with a sensitivity of 97.5%, specificity of 72.0%, and AUC of 0.908. Additionally, a diagnostic panel was established with six miRNAs (miR-151a-5p, miR-200b-5p, miR-30a-3p, miR-100, miR-629, and miR-154-3p) to distinguish lung adenocarcinoma from granuloma, with a sensitivity of 96.0%, specificity of 60.0%, and an AUC of 0.76.

##### Long non-coding RNAs

Long non-coding RNAs (lncRNAs) have shown significant potential because of their stability in biofluids [102, 103], and their frequent dysregulation in the development of NSCLC [104]. Gupta C et al. [105] analysed lncRNA expression in the sputum of lung cancer patients and healthy controls, and reported that a panel consisting of SNHG1, HOTAIR, and H19 could effectively discriminate the two groups, achieving an AUC of 0.90. Similarly, Yuan S. et al. [106] conducted a study involving 528 plasma samples from lung cancer patients, other lung disease patients, and healthy participants. They succeeded in identifying a panel of four lncRNAs (MALAT1, NEAT1, RMRP, and TUG1) that demonstrated strong diagnostic capabilities for NSCLC, with AUC values of 0.85 for adenocarcinoma (AC) and 0.93 for squamous cell carcinoma (SCC) in the validation cohort.

##### Circular RNAs

Circular RNAs (circ-RNAs) can be detected in various biofluids such as plasma, saliva, and exosomes. These genes are aberrantly expressed in early-stage lung adenocarcinoma, indicating their potential as early diagnostic biomarkers for lung cancer. A study conducted by Falin C. et al. [107] confirmed that a specific group of circRNAs (hsa_circ_0001492, hsa_circ_0000690, hsa_circ_0001346, and hsa_circ_0001439) were significantly upregulated in plasma exosomes from AC patients compared with those from healthy individuals. Additionally, three other circRNAs have been evaluated as possible early diagnostic biomarkers for lung cancer through liquid biopsy, demonstrating strong diagnostic accuracy: hsa_circ_0006423(115), hsa_circ_0023179 (114), and circFOXP1 [108].

In summary, utilising ncRNAs as biomarkers for lung cancer screening represents a highly promising approach to incorporating liquid biopsies into preventive strategies. Therefore, it is essential to standardise detection protocols for liquid biopsy ncRNAs and conduct additional prospective clinical trials with larger sample sizes to confirm and implement these innovative biomarkers in clinical practice [109].

##### Autoantibodies

Lung cancers can stimulate the body's immune responses against tumour-associated antigens (TAAs) that are either expressed abnormally or mutated proteins, resulting in the production of antibodies against these TAAs known as autoantibodies (AAbs) [110]. These AABs and their associated antigens may deepen our understanding of cancer immunity, potentially aiding in early diagnosis and enhancing the effectiveness of immunotherapy. Owing to their stability and specificity in serum, TAAbs have gained attention as potential biomarkers and have been the subject of extensive research [111]. Typically, TAAbs persist in serum for extended periods, making them appealing candidates for developing noninvasive blood tests to diagnose or detect cancer early. Nevertheless, while autoantibody panels tend to be specific, they may lack sensitivity [112].

Liu et al. [112] combined TAAbs with CT and demographic characteristics and obtained a diagnostic accuracy of 73.4%, with a specificity of 87.1% and a sensitivity of 61.5%. Wang et al. [110] utilised a dedicated HuProt protein microarray to identify potential TAAbs for detecting lung cancer and distinguishing benign from malignant nodules with an AUC value of 0.845. Mission et al. [113] employed a specific autoantibody-based test (EarlyCDT-Lung) to risk models, enhancing diagnostic performance, with a specificity of over 92% and a positive predictive value exceeding 70%.

##### Metabolic biomarkers

Tracking cancer-related metabolites is a developing and encouraging approach for detecting and diagnosing various types of malignant tumours, such as colorectal, gastric, gynaecological, and lung cancer [114]. In the last twenty years, several metabolomics studies using various biological samples have been conducted via NMR and/or MS techniques to generate metabolite profiles that differentiate lung cancer patients from healthy individuals [115].

Blood samples, such as plasma and serum, are the most frequently analysed biological fluids in lung cancer research. These samples can be utilised to identify metabolic markers through both targeted approaches (which focus on a specific group of compounds) and untargeted approaches (which involve comprehensive global profiling) [116, 117]. Previous research has described changes in amino acid concentrations, particularly alanine, isoleucine, glutamine, histidine, leucine, lysine, and serine concentrations, in the serum or plasma of individuals diagnosed with lung cancer [115].

Lactic acid is another frequently altered metabolite found in lung cancer patients [118, 119]. Phospholipids play crucial roles in the structure of cell membranes [120]. In lung cancer, the metabolic pathways involving phospholipids are often dysregulated, leading to distinct patterns [120].

Klupczynska et al. [121] utilised ultra-high performance liquid chromatography–quadrupole-Orbitrap high-resolution mass spectrometry and identified several potential biomarkers for NSCLC that fall into categories such as amino acids, acylcarnitines, and organic acids. The multivariate ROC curve built from 12 detected metabolites had an AUC of 0.836 (0.722–0.946). Yu et al. [122] performed an extensive lipid profiling study by utilising tandem mass spectrometry and assessed 390 different lipids in the plasma samples taken from early-stage NSCLC patients and healthy controls. They identified a set of four lipid markers—LPE (18:1), C(18:2)CE, SM(22:0), and ePE (40:4), to predict early cancer, achieving an accuracy of 82.3% (AUC), with a sensitivity of 81.9% and a specificity of 70.7% during the training phase. In the validation phase, the predictive performance showed an accuracy (AUC) of 80.8%, with a sensitivity of 78.7% and a specificity of 69.4%.

A significant challenge in metabolomics is the enormous variety and chemical complexity of metabolites, making it difficult for any existing metabolomics method to address these complexities fully [115]. This leads to inaccuracies in the early detection of lung cancer. Therefore, there is a need for a multi-omics approach for the efficient diagnosis of lung cancer.

#### Cell-free DNA

Cell-free DNA (cfDNA) is a term used to describe a collection of circulating DNA fragments present in body fluids. These 140–170 base pair (bp) fragments are released either by normal healthy cells or cancer cells and hence are widely explored as early tumour markers [123–125]. Serum-circulating cell-free DNA (cf-DNA) released from tumour cells is a comprehensive marker for various biological aspects of cancer. Its potential as a tool for early diagnostic and prognostic monitoring in blood-based tests is significant and promising [126]. The detection of cfDNA begins with the proper collection and processing of blood, followed by the isolation and storage of blood plasma. In addition to conventional mutation detection methods, several specialised techniques have been developed to identify low levels of cfDNA amidst an excess of non-mutated DNA. These techniques include real-time polymerase chain reaction (PCR), BEAMing (beads, emulsion, amplification, and magnetics), and denaturing capillary electrophoresis. Enhanced detection of mutations can be achieved through approaches such as mutant-enriched PCR and COLD-PCR (co-amplification at lower denaturation temperature PCR). Furthermore, innovative miniaturised methods, such as single-molecule sequencing, show promise for future advances in cfDNA detection, offering high sensitivity and the potential for comprehensive mutation profiling [127]. Elevated levels of plasma cfDNA have been reported in lung cancer patients compared with healthy controls and people with chronic respiratory inflammation [128].

As expected, cfDNA concentrations are higher in patients with more advanced lung cancer than in patients with early-stage lung cancer. A study performed by Zhou et al. [129] demonstrated a positive correlation between tumour burden and the baseline levels of cfDNA in patients with NSCLC. Even the most sensitive conventional genotyping platforms for detecting cfDNA have a sensitivity of only 70–80% for advanced disease and less than 50% for early-stage disease. This means that a biopsy is still needed even if the results are negative [130]. Importantly, the concentration of cfDNA alone is insufficient as a diagnostic indicator because variations and overlaps are observed between individuals with and without cancer [131].

Considering the difficulties in detecting cfDNA in patients with very small volume disease (stage 1 cancer), researchers have explored cfDNA fragmentation patterns [132, 133]. In this method, whole-genome libraries are developed after the extraction of cfDNA from plasma, upon which low-coverage whole-genome sequencing is employed via machine learning, resulting in significantly improved detection rates for early disease. Different chromatin structures and chromosome compositions in cancer cells compared with normal cells can be explored via the fragment size distribution of cfDNA throughout the genome. These discoveries have led to the DNA evaluation of fragments for the early interception (DELFI) method [134]. Comparing healthy individuals to patients with early-stage cancer, detection rates ranging between 57 and 99% among seven different cancer types have been reported [132].

A series of DELFI studies have been conducted or commenced, starting with DELFI-L101, which was completed in 2023. Individuals at risk of developing lung cancer (per the existing screening guideline recommendations) had blood testing performed [135]. Following that study, FirstLook Lung, a cfDNA blood-based test specifically for lung cancer screening, is now commercially available. This is the only such commercially available test in the United States [136]. Currently, two major prospective case–control studies are in progress for the early detection of lung cancer in screening populations: DELFI-L201/NCT05306288 and DELFI-L301/NCT06145750.

#### DNA methylation

DNA methylation patterns change during tumorigenesis; therefore, tumour-specific methylation patterns are potential biomarkers that indicate malignancy. DNA methylation involves enzymatic modification of DNA bases in mammalian cells, accomplished by adding a methyl group to the 5th carbon atom of cytosine (5mC) [137]. Regions with a greater percentage of GpG dinucleotides (greater than 55%) are called CpG islands. CpG islands are sequences close to or within gene promotor regions, often including a gene’s first exon and/or intron. Methylation at CpG sites is catalysed by DNA methyltransferase (DNMT) proteins. Aberrant DNA methylation has been shown to negatively affect gene expression [138]. However, DNA methylation does not alter the DNA sequence but instead silences genes to which the methyl group is attached [139].

Understanding how CpG island methylation causes the inactivation of tumour suppressor genes is crucial [140]. The methylation levels in the serum samples were significantly greater for patients with malignant nodules than for those with benign nodules. Higher methylation rates (65.5% and 67.2%) were observed for the serum RUNX3 and RASSF1A genes in patients with malignant nodules than in those with benign nodules (12.3% and 10.1%, respectively) [141]. One study used cfMeDIP-seq methylation profiling to establish a prediction model to efficiently differentiate malignant pulmonary nodules from normal controls with high sensitivity (91%), specificity (93%), and AUC (0.963) [142]. Liang et al. [143] developed a blood-based DNA methylation panel, termed "PulmoSeek", using 140 patient plasma samples (100 malignant and 40 benign samples). They achieved an overall AUC value of 0.843 and a high sensitivity of 0.99 when lung nodules were triaged. Despite the excellent accuracy and sensitivity, the study showed a poor specificity of 0.325. To address this limitation, the same group developed "PulmoSeek Plus", a panel that combines DNA methylation and clinical features via ML techniques and found an improved specificity of 0.5, a sensitivity of 0.99, and an AUC of 0.90 [144].

Although blood-based panels are widely used, increasing attention has been given in recent years to exploring other body fluids, such as bronchoalveolar lavage fluid (BALF). A study indicated that DNA methylation from genes sourced from BALF demonstrated superior performance compared with DNA methylation derived from plasma [131]. BALF methylation achieved an overall accuracy of 0.813, sensitivity, and specificity of 0.81, whereas plasma methylation achieved an accuracy of 0.688, sensitivity of 0.667, and specificity of 0.714 [131]. In another study, Li et al. [145] identified the diagnostic potential of eleven lung cancer-specific DNA methylation markers (CDO1, GSHR, HOXA11, HOXB4-1, HOXB4-2, HOXB4-3, HOXB4-4, LHX9, MIR196A1, PTGER4-1, and PTGER4-2) for accurately differentiating benign from malignant nodules. They achieved a specificity of 0.82, a sensitivity of 0.7 and an AUC of 0.82. In another study, integrating the methylation of four genes (SOX17, HOXA7, CDO1, and TAC1) achieved a specificity of 0.62, a sensitivity of 0.93 and an AUC of 0.77 for triaging lung nodules [146]. Notably, the DNA methylation levels in the sputum samples exceeded those in the plasma samples, with a specificity of 0.71, a sensitivity of 0.98 and an AUC of 0.89. Furthermore, by combining clinical data with DNA methylation data derived from sputum samples, the diagnostic performance (AUC of 0.91) improved [146]. Table 1 summarises previously published studies that have used DNA methylation as a biomarker to triage lung nodules. A study from Guangzhou Medical University (NCT03181490) evaluated the potential of a ctDNA assay for differentiating benign and malignant pulmonary nodules via targeted high-throughput DNA methylation sequencing [147].

**Table 1:** DNA methylation as a biomarker for the differential diagnosis of malignant lung nodules from benign lung nodules

Year	Sample types	Nodule/Lesion Size	No. of patients N = total, M = malignant, B = Benign, C = control	Independent training set and a validation set—study design	Sensitivity	Specificity	AUC	Genes	Ref
2021	Tissue and BALF	N/A	Tissue:*N* = 57, M = 28, B = 29) BALF samples (*N* = 181,M = 95, B = 86)	Yes	Test set: 0.82 Validation set: 0.70	Test set: 0.91 Validation set: 0.82	Test set: 0.93 Validation set: 0.82	11 genes	[145]
2021	Plasma		M = 67(35 < 30 mm,32 > 30 mm) B = 23,C = 7	Yes	0.91	0.933	0.963	300 DNA methylation regions	[142]
2021	Tissue and (BALF)	≤ 20 mm	*N* = 53, M = 32, B = 21	No	BALF panel: 0.81 Plasma panel: 0.667	BALF: 0.81 Plasma: 0.714	BALF: 0.82 Plasma: 0.68	168 lung cancer-related genes	[131]
2021	Plasma	6 to 20 mm	*N* = 529,M = 116, B = 413	Yes	Overall: 0.990 Test set: 0.933	Test set: 0.6 Overall: 0.325	0.843	100 methylated genes	[143]
2021	Plasma	N/A	*N* = 500 patients M = 214, B = 264 C = 20	Yes	0.87	0.98	0.938	SHOX2, RASSF1A, and PTGER4	[149]
2020	Plasma	≤ 30 mm	M = 163 malignant lung nodules B = 83	Yes	Combination of three genes: 0.90	Combination of three genes: 0.71	Combination: 0.88	CDO1, TAC1, SOX17, HOXA7, HOXA9, GATA4, GATA5, and PAX5	[150]
2020	Plasma, Tissue	Stage I, II, III, IV)	*N* = 140 M = 104, B = 36	Yes	Total methylation Training set: 0.654 Validation set: 0.684	Training set: 0.971 Validation set: 0.919	0.8613	SHOX2 and PTGER4	[151]
2019	Learning set: Tissue Training set: Plasma	< 30 mm	*N* = 309. M = 168, B = 139	Yes	Learning set: 0.927 Validation set: 0.795	Learning set: 0.928 Validation set: 0.852	Learning set: 0.974 Training set: 0.839 Validation set: 0.816	13 CpG sites of TCGA database:	[152]

##### DNA methylation sample combinations

A separate study explored the combined advantages of using sputum and plasma as potential biomarkers by analysing DNA methylation. This research included a DNA methylation gene panel study of 210 patients with nodules, comprising 150 patients with malignancies and 60 controls. The AUC was 0.89 for sputum and 0.77 for plasma. However, combining sputum testing with clinical data correctly predicted lung cancer in 91% of the subjects, whereas the prediction was 85% accurate when plasma testing was combined with clinical data [146]. In a similar 2021 study, researchers assessed plasma DNA methylation in 220 patients, yielding an AUC of 0.91 in the training set. When specific high-risk clinical features were included in a validation cohort, the AUC increased to 0.948 [148].

#### Circulating tumour cells

Circulating tumour cells (CTCs) are tumour cells that disseminate from malignant lesions. CTCs detach from primary, secondary and/or metastatic sites and enter the lymphatic system or the bloodstream [153]. Since CTCs can be detected prior to imaging and clinical symptoms, CTCs are highly relevant as biomarkers [154, 155]. CTCs intravasate into the bloodstream by penetrating the basal membrane through a process termed epithelial‒mesenchymal transformation (EMT) [155–157]. CTCs can be detected as single cells or as homotypic or heterotypic clusters with white blood cells (WBCs) in circulation [158–160]. However, detecting these CTCs is challenging due to the low number of CTCs in a blood sample, especially in the early stages of cancer [161]. Current methods for CTC isolation and enrichment include (1) cell size or density (biophysical properties), (2) immunoaffinity (cell surface markers) [157], and (3) positive and negative enrichment [162]. The CELLSEARCH® system is the only FDA-approved CTC enrichment platform for counting CTCs for clinical use [163].

Numerous clinical studies have revealed the association between CTC numbers and the potential to differentiate between malignant and benign nodules (Table 2) [164]. Detecting CTCs in blood samples from patients with malignant lesions can improve diagnostic accuracy and prevent ethically unacceptable repeat tissue biopsies [165, 166]. A prospective study involving 221 patients revealed that CTCs and tumour-macrophage fusion (TMF) cells could complement LDCT, aiding in differentiating malignant and benign lung nodules [167]. While studies have identified CTCs in patients with both malignant and benign lung nodules, those with benign nodules typically exhibit lower CTC numbers than patients with malignant nodules do [168, 169].

**Table 2:** Circulating tumour cells as biomarkers for the differential diagnosis of lung nodules

Year	Nodule Size	No. of patients *N* = total, M = malignant, B = Benign, C = control	Biomarker	Independent Training set and Validation set study design	Sensitivity	Specificity	AUC	Detection and Staining Technique	Ref
2022	Lung-RADS 4	*N* = 221	Single CTCs, CTC clusters, TMF	Yes	0.87	1	0.989	IHC:CK^+^/EpCAM^+^/CD14/CD45^–^ with a DAPI^+^ nucleus	[167]
2022	> 8 mm	*N* = 538, M = 282,B = 256	FR + CTC	Yes	0.819	0.8086	0.828	Negative enrichment and ligand-targeted PCR assays	[169]
2021	≤ 30 mm	*N* = 24	CEP8 + CTCs	No	0.75	1	N/A	CEP8 CTC detection kit (Cyttel®, Cyttel Bio), based on the CD45-FISH technique, IHC: CTCs (DAPI + /CD45 − /CEP8 +	[171]
2021	Stage0-III	*N* = 53	CEP8 + CTCs	No	0.927	0.5	0.713	Cyttel method (Cyttel, Jiangsu, China) IHC: CEP8 + /CD45-/DAPI +	[172]
2020	> 6 mm + Stage I, II, III, IV	*N* = 207, M = 107,C = 100	CTC	Yes	0.89	1	0.942	Antigen-independent, 4-color (FISH)- based method	[173]
2020	> 5 mm + Stage I, II, III, IV	*N* = 614	CTC	No	0.263	0.962	N/A	ISET Rarecells (Rarecells Diagnostics, Paris, France)	[174]
2020	N/A	*N* = 3798	FR + CTC	No	0.87	N/A	N/A	Leukocyte-based negative enrichment strategy	[175]
2019	Stage I-IV	*N* = 80 patients, M = 29, B = 31, C = 20	CTC clusters	No	0.41	N/A	N/A	IHC: Cytokeratins (CK) 8/18 and/or 19, EpCAM, CD45, and nuclei were stained with DAPI	[168]
2019	Tumour staging (i.e., I, II, III, IV)	M = 174B = 37, C = 53	CTC	No	0.6839	1	0.846	Negative enrichment–fluorescence *in situ* hybridisation (NE-FISH) IHC: DAPI + /CD45 − /CEP 8 +	[176]
2019	≤ 30 mm	*N* = 382	FR + CTC	Yes	0.786–0.827	0.688–0.784	Trainingset: 0.781 Validation set: 0.792	CytoploRare® Detection Kit provided by GenoBiotech (China) Co. Ltd IHC: CD45, FRα, and DAPI	[177]
2018	Tumour staging (i.e., I, II, III, IV) > 30 mm vs < 30 mm	M = 72, b = 24, C = 2	CTC	No	0.8194	0.7308	0.8221	CytoploRare® FR-positive cell detection kit (Genosaber, Shanghai, China)	[166]
2017	< 5 mm to > 10 mm	M = 47 C = 13	CTC	No	0.73	N/A	N/A	Cell collector was coated with anti-EpCAM antibodies IHC: EpCAM/CK, CD45 and DAPI/Hoechst	[178]
2017		M = 75	CTC, CTM	No	0.701	1	N/A	ScreenCell Cyto filtration devices Staining with hematoxylin solution S (Merck KGaA)	[179]
2021	< 20 mm	*N* = 120,M = 89,B = 31	CTCs	No	0.899	0.839	0.843	Telomerase reverse transcriptase–based (TERT-based) CTC detection	[180]

Abbreviations: *CD45* cluster of differentiation 45, *CEA* Carcinoembryonic antigen, *CK* cytokeratin, *CTC* circulating tumour cells, *CTM* circulating tumour microemboli, *CEP8* Chromosome 8 centromere detection probe, *DAPI* 4',6-diamidino-2-phenylindole, *EpCAM* epithelial cell adhesion molecule, *FR* + *CTC* FR-positive circulating tumour cell, *ISET* Isolation by Size of Epithelial Tumour cells, *IHC* Immunohistochemistry, *SE-iFISH* single-end *in situ* hybridisation fluorescence *in situ* hybridisation, *TMF* tumour-macrophage fusion cells

In general, CTCs hold great promise as true positive components for nodule evaluation. It is remarkable to consider how early these cells enter the circulation from small tumours. However, they are currently unlikely to be used to rule out cancer in a nodule; that is, they can not reliably be used as true negatives in early-stage lung cancer.

Liquid biopsy studies face significant challenges, particularly in standardisation during the preanalytical phase and sample biobanking. Preanalytical factors critically impact the reproducibility and accuracy of biomarker detection and quantification. Addressing variability in sample handling—such as collection, isolation, and short-term storage—is crucial for achieving consistent, reliable, and reproducible results. The regulatory, ethical, and economic obstacles that necessitate careful consideration highlight the urgent need for standardisation in this field [170].

### Breath-based biomarkers for triaging lung nodules

The term 'breathomics' refers to metabolites in exhaled air generated through cellular biochemical reactions. Breathomics testing has emerged as a promising approach for detecting and screening for lung cancer, offering a potential approach to predict the onset of the disease even before clinical symptoms become evident. This approach provides several benefits, including noninvasiveness, user-friendliness, cost-effectiveness, and long-term monitoring. Compared with blood or urine sampling, exhaled breath has a simpler composition, allowing for direct analysis without complex sample preparation [181].

#### Volatile organic compounds(VOCs)

VOCs have been widely studied as biomarkers of advanced lung cancer [182]. There are also reports of the high sensitivity of VOC detection for very early-stage lung cancer [183]. VOCs are byproducts of various biological processes, including cell injury, death, oxidative stress, and inflammation [184]. VOCs produced by cellular metabolism are released into the bloodstream and excreted through exhaled breath or body fluids. As cancer progresses, alterations in the genome and transcriptome result in disturbances in metabolic pathways and the accumulation of abnormal metabolites in breath [185]. Nonmalignant processes can alter VOC patterns due to food consumption, medications, smoking and noncancer diseases such as diabetes or chronic renal failure [186].

Gas chromatography‒mass spectrometry (GC–MS) is currently considered the most reliable technique for analysing human breath biomarkers [185]. Alternative methods for analysing breath include solid-state pattern recognition devices (electronic noses), which can be trained to detect patterns consistent with malignancy but are unable to identify specific VOCs [187]. Chen et al. [188] reported a promising miniature eNose system: they combined 14 gas sensors from 4 different sensor array types. They studied 134 controls and 101 lung cancer cases (a range of clinical stages). They applied neural network AI to their results and achieved a 90% diagnostic accuracy. These authors commented upon how their results could lead to a low-cost bespoke, designed eNose system for community use. However, larger clinical trials are needed.

Using GC–MS, many studies have identified VOCs specific to lung cancer patients by comparing breath samples from lung cancer patients to those from healthy individuals or individuals with benign lung nodules [189]. However, as summarised in Table 3, the results of these studies vary significantly due to differences in sample collection methods, patient characteristics, and testing environments [190]. There is currently a significant disparity between the research conducted on breathomics for lung cancer detection and its practical implementation in a clinical setting. However, further research is necessary to investigate the mechanisms underlying the identification of VOCs as potential markers for lung cancer [190].

**Table 3:** Volatile organic compounds as biomarkers for differentiating benign nodules from malignant lung nodules

Year	Nodule size/stage	No. of Patients *N* = total M = malignant B = benign nodule C = controls	Independent Training set and Validation set study design	Sensitivity	Specificity	AUC	Biomarker	Technology	Ref
2021	Stage I (36.4%) 56 II (11.7%) 18 III (14.3%) 22 IV (37.7%) 58	*N* = 352, M = 160; B = 70, C = 122	Yes	N/A	N/A	BPN from LC: 0.809	17 VOCs	TD-GC–MS	[196]
2019	LDCT screen-detected nodules < 3 cm	*N* = 301, including biopsy-proven malignant nodules	Yes	0.8	0.75	0.8	8 VOCs	GC MS	[197]
2017	31.6 ± 15.0 mm (for malignant nodules)	*N* = −119, M = 89, B = 30	Yes	0.75	0.933	0.87	40 VOCs	Nanomaterial-based sensor array	[198]

A significant challenge in this emerging area of breathomics is the correlation of volatile compounds detected in breath with the associated (patho)physiological processes. Notably, the data from clinical trials utilising human breath are not yet adequately integrated with our understanding of functional and mechanistic physiology [191].

Patients with COPD are typically middle-aged or elderly, and they often experience a loss of skeletal muscle as their disease progresses. Consequently, the exhaled levels of certain volatile organic compounds (VOCs) influenced by both age and metabolism—such as isoprene, acetone, and alkanes—may be affected not only by the disease itself but also by the natural aging process. Isoprene and acetone are present at relatively high concentrations in exhaled breath compared with other VOCs, thus contributing significantly to the nonspecific eNose pattern [192].

eNose systems also encounter various challenges, primarily related to the nature of the sensors used in these devices [193]. One critical issue is their difficulty in discriminating and quantifying odours at very low concentrations. Another significant challenge is the response characteristics of the sensors that make up the eNose systems. Notably, significant sensor drift can occur, particularly when exposed repeatedly to gas mixtures over short periods or under high humidity conditions and sudden temperature fluctuations. These challenges may result in inaccurate diagnostics, undermining the reliability of the data produced by the eNose [187].

Other practical challenges include the possible effects of subjects’ recent consumption of food (which can affect VOC profiles) and the duration and type of fasting prior to testing [194, 195].

## Biomarker combination studies

### cfDNA combinations

An important novel study recently reported the next phase of Pulmoseek cfDNA investigations: Pulmseek PLUS [147]. This was a large prospective study using a combined approach of the previously mentioned analysis of cfDNA methylation testing (Pulmoseek) along with clinical and imaging biomarkers (CIBMs). The CIBM model was first validated in 2 cohorts with over 500 patients. This was then combined with Pulmoseek in two cohorts of 258 and 283 patients respectively. Overall, the Pulmoseek Plus had a better performance, increasing the AUC from 0.85 for the separate models to 0.90 for the combined model. It had an overall sensitivity of 0.98 (097–0.99) and a fixed specificity of 0.50 for ruling out cancer. This was still excellent for nodules of 5–10 mm in size, with a sensitivity of 0.99. Very importantly, there were significant demonstrable benefits in terms of treatment and biopsy decision-making; if Pulmoseek Plus with a range of cut-off points was used, it was possible to potentially prevent 89% of unnecessary surgeries (105 of 118 such cases) and 73% of instances of delayed treatments (308 of 423 such cases).

In a 2021 study [199] involving 98 high-risk patients, researchers compared the effectiveness of using plasma cfDNA methylation alone to the combined use of this marker with clinical features, cell-free DNA mutations, and protein cancer biomarkers. Among the nodules examined, 70 were malignant, whereas 28 were benign. An independent validation cohort was further performed with another 29 nodules, and it was found that methylation alone had an AUC of 0.72 in the validation set. Adding the other markers led to training and validation set area under the curve values of 0.85 and 0.86, respectively.

A combination of cfDNA with CT screening is being evaluated via the DELFI diagnostic blood test as previously mentioned in a new study: the 4-IN-THE-LUNG-RUN European lung cancer screening study [136]. This study aimed to evaluate the performance of this blood test in patients with a negative initial CT screening scan for lung cancer. It aims to recruit some 9000 subjects. The analysis will determine which patients should benefit from repeat CT scans.

### CTC combinations

CTCs were combined with serum CEA by Zheng et al. [165] using a cohort of 85 patients with malignant nodules with a mean diameter of 17 mm and 46 patients with benign nodules. CTCs were analysed via subtraction enrichment, immunostaining, and fluorescence *in situ* hybridisation (SE i-FISH) method to determine the gene copy numbers of eight chromosomes and the CK18 tumour antigen. CTC counts were clearly higher in malignant cases (*p* = 0.016) (sensitivity of 67.1% and specificity of 56.5%), especially in the upper lobes, but were unrelated to tumour size. There was an improvement when serum CEA and nodule type (solid versus GGO) were added: the combined AUC was 0.827 (CI 0.752–0.901), indicating satisfactory discrimination of patients with early-stage NSCLC from those with benign nodules.

Another study of 726 patients in 12 tertiary hospitals integrated clinical model parameters (age and smoking history), observable characteristics of nodules (size, count, location, attenuation), and an AI assessment of LDCT scans [200]. The AI platform was “trained” on 20,000 CT nodules with known histopathological diagnoses. Three-dimensional deep convolutional neural networks were developed to create a malignant/benign nodule classifier, which was then used to analyse peripheral blood samples for malignant cells. They also performed a blood-based four-colour FISH assay for four DNA probes that are universally deleted in NSCLC and have been implicated in the tumour pathogenesis, with an AUC of 0.74 for the AI assessment and an AUC of 0.765 for FISH testing. In addition to AI and FISH, a series of parameters were developed from the training cohort, which included the clinical characteristics (age and smoking status), radiological characteristics (diameter, nodule count, subsolid status, upper lobe location, and malignant signs at the nodule edge), AI risk score and liquid biopsy results from a 4-color FISH assay. The final AUC in the independent validation cohort of 168 patients was 0.895. This integrated model achieved a sensitivity of 82.86% (95% CI: 74.27–89.51%) and a specificity of 80.95% (95% CI: 69.09–89.75%) for distinguishing between malignant and benign nodules. In a 2021 study involving 234 patients, circulating tumour cells (CTCs) were combined with radiomic features. The AUC for CTCs alone was 0.72, but it increased by 2.5 points when CTCs were combined with radiomic features [201]. A 2022 study involving 224 patients explored the combination of CTC counts and serum tumour marker levels. The AUC for CTCs alone was 0.81. However, the combination of CTC counts and serum tumour levels had a high AUC value of 0.853 [164].

### Breath combinations

Shaffie et al. [202] reported an AI nodule evaluation combined with breath testing in 47 patients, 37 with malignant nodules and 10 with benign nodules. Spherical Harmonic-based shape features were used to quantify the pulmonary nodules and the nodules' volumetric features (size). VOCs in exhaled breath (27 were tested) were quantified via mass spectrometry. A deep-learning autoencoder classifier using CT and VOC data was developed for nodule classification. Additional retrospective CT scans of 727 pulmonary nodules and breath samples from 504 patients were analysed to develop the model. When applied to the 47 patients, this combined data analysis tool achieved 97.8% accuracy, 97.3% sensitivity, 100% specificity, and 99.1% AUC when classifying malignant nodules from benign nodules.

## Future perspectives

Our review highlights the importance of integrating biomarkers from body fluids and breath analysis with AI-driven assessments of lung nodules to enhance the accuracy of detecting malignant lung nodules. These novel, minimally invasive methods have great potential to transform the clinical management of lung nodules by increasing patient outcomes. Furthermore, liquid biopsy biomarkers such as microRNAs, exosomes, and proteomes show great potential for improving the molecular triage of benign versus malignant nodules, potentially addressing issues of tissue heterogeneity. However, studies involving liquid biopsies encounter challenges related to the standardisation of sample handling and storage, which can affect the accuracy of biomarkers. Additionally, cfDNA holds promise for early cancer diagnosis but requires advanced detection techniques. Radiomics struggles with issues such as model reproducibility and data quality, limiting its clinical use. The eNnose systems involve sensor calibration and the ability to detect low concentrations of volatile compounds, which in turn affects diagnostic reliability. Additionally, the correlation between volatile compounds in breath and physiological processes requires further investigation and understanding. To address the various challenges discussed in this review, future research should focus on improving imaging technologies, developing better risk prediction models, refining AI algorithms, and promoting multidisciplinary approaches involving radiologists, pulmonologists, and oncologists for lung nodule evaluation. Future research and development strategies in this field should focus on expediting the differential diagnosis of lung nodules and facilitating the transition from laboratory to clinical settings. This will significantly reduce the need for invasive procedures and enhance patient outcomes. Ultimately, the biomarkers and combinations discussed in this review require further validation through large multicenter clinical trials before they can be implemented in routine clinical practice.

## Data Availability

Not relevant

## Declarations

### Permission to reproduce material from other sources

None of the figures were taken from other people’s work. The figures were prepared using BioRender software.
